# *Haematococcus pluvialis* extract YHC-T-2414 mitigates exercise- induced fatigue by promoting antioxidant defense and mitochondrial biogenesis

**DOI:** 10.3389/fnut.2026.1906109

**Published:** 2026-08-12

**Authors:** Jeongho Jeong, Hyejin Jeon, Aeri Song, Hyun-Je Park, Junghyun Kim

**Affiliations:** 1 Yuhan Care Co., Ltd, R&D Center, Hwaseong, Republic of Korea; 2 Department of Oral Pathology, School of Dentistry, Jeonbuk National University, Jeonju, Republic of Korea; 3 Non-Clinical Evaluation Center, Biomedical Research Institute, Jeonbuk National University Hospital, Jeonju, Republic of Korea

**Keywords:** anti-fatigue effects, antioxidant effects, C2C12 myoblasts, *Haematococcus pluvialis* extract, mitochondrial biogenesis

## Abstract

Algae have long been used for nutritional and health-promoting purposes in several cultures. *Haematococcus pluvialis*, a microalga widely recognized as a natural source of astaxanthin, has attracted growing interest as a functional bioresource with potential to improve physical performance and alleviate fatigue; however, its underlying mechanisms remain unclear. This study aimed to evaluate the anti-fatigue effects of *H. pluvialis* extract (YHC-T-2414) and its underlying mechanisms. The effects of YHC-T-2414 on cell viability, and lactate dehydrogenase (LDH) and creatine kinase (CK) activities were evaluated in hydrogen peroxide (H_2_O_2_)-induced C2C12 myoblasts. Its antioxidant effect was assessed by measuring reactive oxygen species (ROS) generation. Mice were administered YHC-T-2414 for 3 weeks and subjected to an exhaustive swimming test. Subsequently, fatigue-related biochemical parameters, including lactate, LDH, CK, malondialdehyde (MDA), tumor necrosis factor-alpha (TNF-α), and interleukin-6 (IL-6) were measured in serum and muscle tissues. YHC-T-2414 attenuated H_2_O_2_-induced reductions in cell viability, and reduced LDH and CK activities and ROS generation in H_2_O_2_-treated C2C12 myoblasts. Furthermore, it significantly increased swimming time in the forced swimming mouse model while reducing lactate, LDH, CK, MDA, TNF-α, and IL-6. Notably, the anti-fatigue effects of YHC-T-2414 may be associated with regulation of the AMPK-NRF2-KEAP1 and AMPK-SIRT1-PGC-1α signaling pathways. Overall, YHC-T-2414 may alleviate fatigue by enhancing antioxidant responses and supporting mitochondrial biogenesis-related signaling pathways.

## 1 Introduction

Fatigue is a physiological state involving both physical and mental exhaustion induced by prolonged or high-intensity physical activity and daily stress, and it has become a prevalent health concern in modern society (1). Although fatigue often lacks specific clinical symptoms, making it easily overlooked, prolonged neglect can lead to chronic fatigue (2). Chronic fatigue is defined as a sustained state of exhaustion lasting beyond 6 months that remains unresponsive to rest. It is not regarded as a single disease but rather as a syndrome frequently accompanied by various comorbid conditions, including fibromyalgia, irritable bowel syndrome, headaches, and endocrine abnormalities (3). Accordingly, fatigue has become a growing medical and social concern in recent years. However, despite the use of pharmacological agents, nutritional supplements, and behavioral interventions for its treatment and management, their therapeutic efficacy remains insufficient (4). Therefore, identifying effective and safe agents that improve fatigue by restoring physiological balance is essential.

A wide range of factors contribute to fatigue development, with excessive oxidative stress accumulation proposed as a major contributor. Previous studies have demonstrated that reactive oxygen species (ROS) accumulation in the body promotes oxidative stress–mediated DNA damage and apoptosis in C2C12 cells (5) and adversely affects mitochondrial function (6). ROS can interact with cellular components, including lipids, proteins, and nucleic acids. When present in excess, they promote lipid peroxidation in membrane structures, resulting in skeletal muscle damage (7). Therefore, suppression of oxidative stress through the regulation of ROS and malondialdehyde (MDA) levels can be considered a critical mechanism for fatigue alleviation (8). AMP-activated protein kinase (AMPK), a key cellular energy sensor, is activated under conditions of energetic stress, such as prolonged or intense exercise, and plays a central role in maintaining cellular energy homeostasis. Activated AMPK enhances antioxidant defense by promoting nuclear factor erythroid 2-related factor 2 (NRF2)-kelch-like ECH-associated protein 1 (KEAP1) signaling pathway, which induces the expression of antioxidant enzymes and protects cells against oxidative stress (9). In addition, AMPK promotes mitochondrial biogenesis through activation of the sirtuin 1 (SIRT1)-peroxisome proliferator-activated receptor gamma coactivator-1 alpha (PGC-1α) signaling pathway, thereby improving mitochondrial function and energy metabolism (10, 11). Mitochondria are essential organelles responsible for adenosine triphosphate (ATP) production, required for physical activity (12). Mitochondrial dysfunction results in reduced ATP production and a sustained energy-deficient state, which can lead to physical fatigue, thereby positioning mitochondrial biogenesis as a key mechanism for fatigue alleviation (13). Collectively, these findings suggest that activation of AMPK coordinates both antioxidant defense and mitochondrial biogenesis, making the AMPK-KEAP1 and AMPK-SIRT1-PGC-1α signaling pathways promising therapeutic targets for alleviating exercise-induced fatigue.

*Haematococcus pluvialis* is a freshwater microalga that has attracted considerable attention as a functional natural resource because of its diverse biological activities. *H. pluvialis* has been reported to exhibit a range of biological activities, including antioxidant, anti-inflammatory, and anticancer effects (14). Astaxanthin, a carotenoid predominantly accumulated in *H. pluvialis*, has been widely investigated as a bioactive compound with potent antioxidant properties and potential health-promoting effects. Recent studies have highlighted the potential of astaxanthin and other algae-derived bioactive compounds as functional nutraceutical ingredients (15). Previous studies have demonstrated the potential anti-fatigue effects of *H. pluvialis* in improving physical fatigue in both animal models and human clinical trials (16, 17). However, most previous studies have focused on purified astaxanthin rather than the whole *H. pluvialis* extract and have primarily reported physiological outcomes without elucidating the underlying molecular mechanisms. In particular, it remains unclear whether the anti-fatigue effects of the whole extract are associated with coordinated regulation of antioxidant defense and mitochondrial biogenesis through the AMPK-NRF2-KEAP1 and AMPK-SIRT1-PGC-1α signaling pathways.

Therefore, this study aimed to evaluate the anti-fatigue effects of *H. pluvialis* extract (YHC-T-2414) using C2C12 myoblasts and a weight-loaded forced swimming mouse model. Given the limited mechanistic evidence regarding the anti-fatigue potential of *H. pluvialis* extract itself, this study focused on elucidating its antioxidant effects mediated by the AMPK-NRF2-KEAP1 pathway and its role in enhancing mitochondrial biogenesis via the AMPK-SIRT1-PGC-1α pathway. Furthermore, we evaluated the effects of YHC-T-2414 on physical exercise performance and fatigue-related blood biomarkers. Collectively, this study provides mechanistic insight into the anti-fatigue properties of YHC-T-2414 and supports its potential as a natural anti-fatigue agent.

## 2 Materials and methods

### 2.1 Preparation of YHC-T-2414 and determination of astaxanthin

**FIGURE 1:**
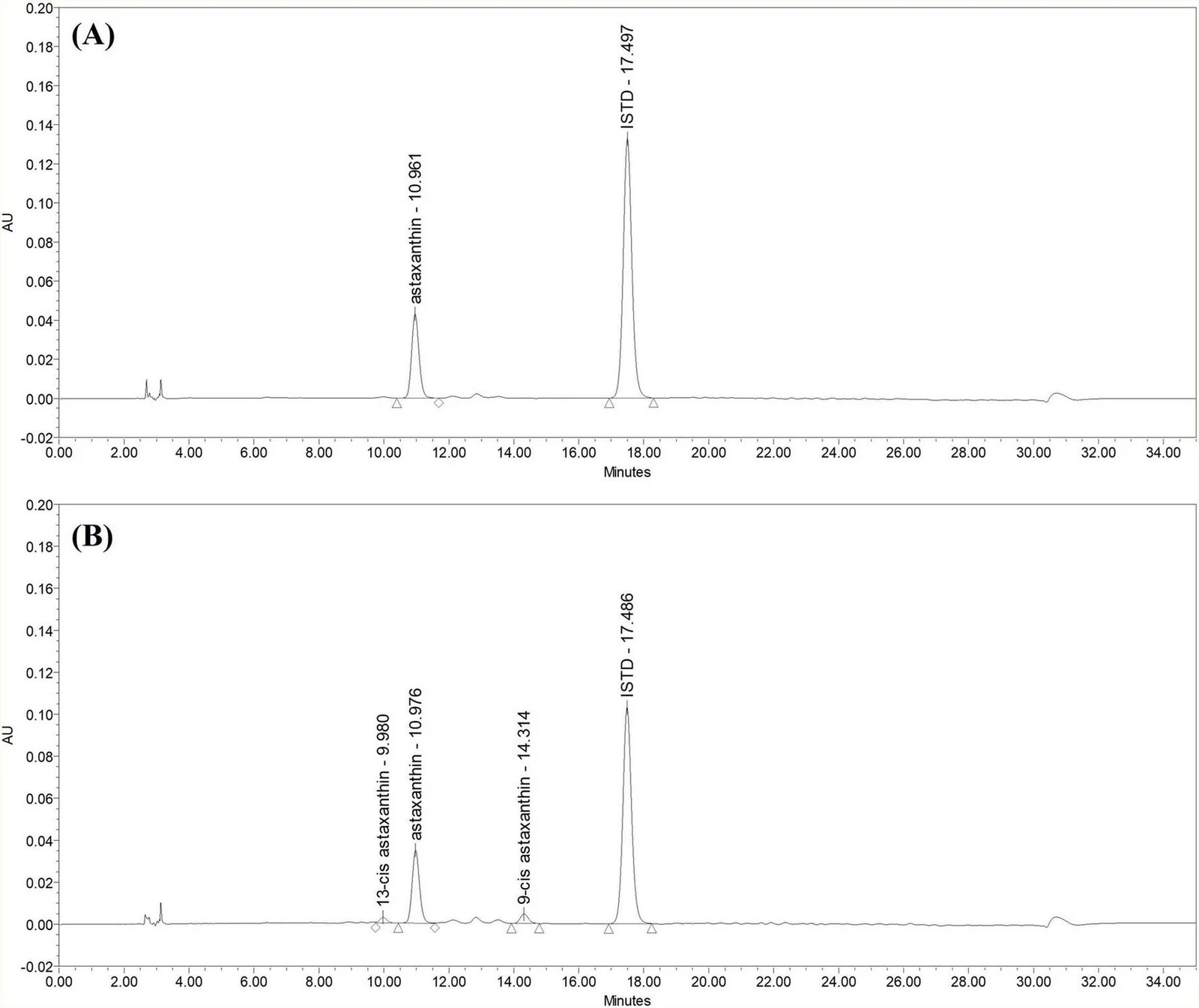
Composition profiles of astaxanthin in YHC-T-2414. HPLC chromatograms of **(A)** astaxanthin standard and **(B)** YHC-T-2414 detected at 474 nm. YHC-T-2414 contained an astaxanthin concentration of 100 mg/g. HPLC, high-performance liquid chromatography.

Dried biomass of *H. pluvialis* was purchased from AlgaeBio Co. (Asan, Republic of Korea). The material was authenticated by Professor Hyoun Woong Shin, and a voucher specimen (SCH-2024-HP01) was deposited in the Department of Life Science, Soonchunhyang University, Asan, Republic of Korea. The dried biomass of *H. pluvialis* was extracted using a supercritical carbon dioxide (CO_2_) extraction system, in accordance with the manufacturing standards specified in the Health Functional Food Code issued by Ministry of Food and Drug Safety (Republic of Korea). The resulting *H. pluvialis* extract, designated YHC-T-2414, was subjected to quantitative analysis to determine its active component profile. Astaxanthin concentration was determined using high-performance liquid chromatography (HPLC) according to the standardized protocol of the Health Functional Food Code (Method 3-45-1: Astaxanthin, Method 1). The results confirmed that the YHC-T-2414 contained 100 ± 5 mg/g of total astaxanthin (sum of trans- and cis-isomers) (Figure 1).

### 2.2 Quantitative analysis of YHC-T-2414

The total astaxanthin content was determined via HPLC according to the Health Functional Food Code (Method 3-45-1). The analysis accounted for individual isomers using specific conversion factors and relative retention times (RT).

### 2.3 Calculation formulas

The total concentration (*C*,μ*g*/*mL*) was derived from the absorbance at 474 nm:


$$\text{C}=\frac{\text{Absorbance}\times 10,000}{2,100}$$

To quantify the total content (%), the peak area of *trans*-astaxanthin and its *cis*-isomers was adjusted by their respective conversion factors (1.3 for 13-*cis*; 1.1 for 9-*cis*) relative to the internal standard (*P*_*i.s*_):


$$\text{Area}\,\text{Ratio}=\frac{{\text{P}}_{\text{trans}}+\left(1.3\times {\text{P}}_{13-\mathrm{cis}}\right)+\left(1.1\times {\text{P}}_{9-\mathrm{cis}}\right)}{{\text{P}}_{i.s}}$$

$$\text{Total}\text{Astaxanthin}\left(\%\right)=\frac{C\times a\times b}{S\times 10,000}$$

(where a : total volume, b : dilution factor, S : sample weight)

Isomers were identified by their relative RTs compared to the internal standard: 13-*cis*-astaxanthin: 0.545, *trans*-astaxanthin: 0.606, 9-*cis*-astaxanthin: 0.800.

### 2.4 Cell culture and viability

**FIGURE 2:**
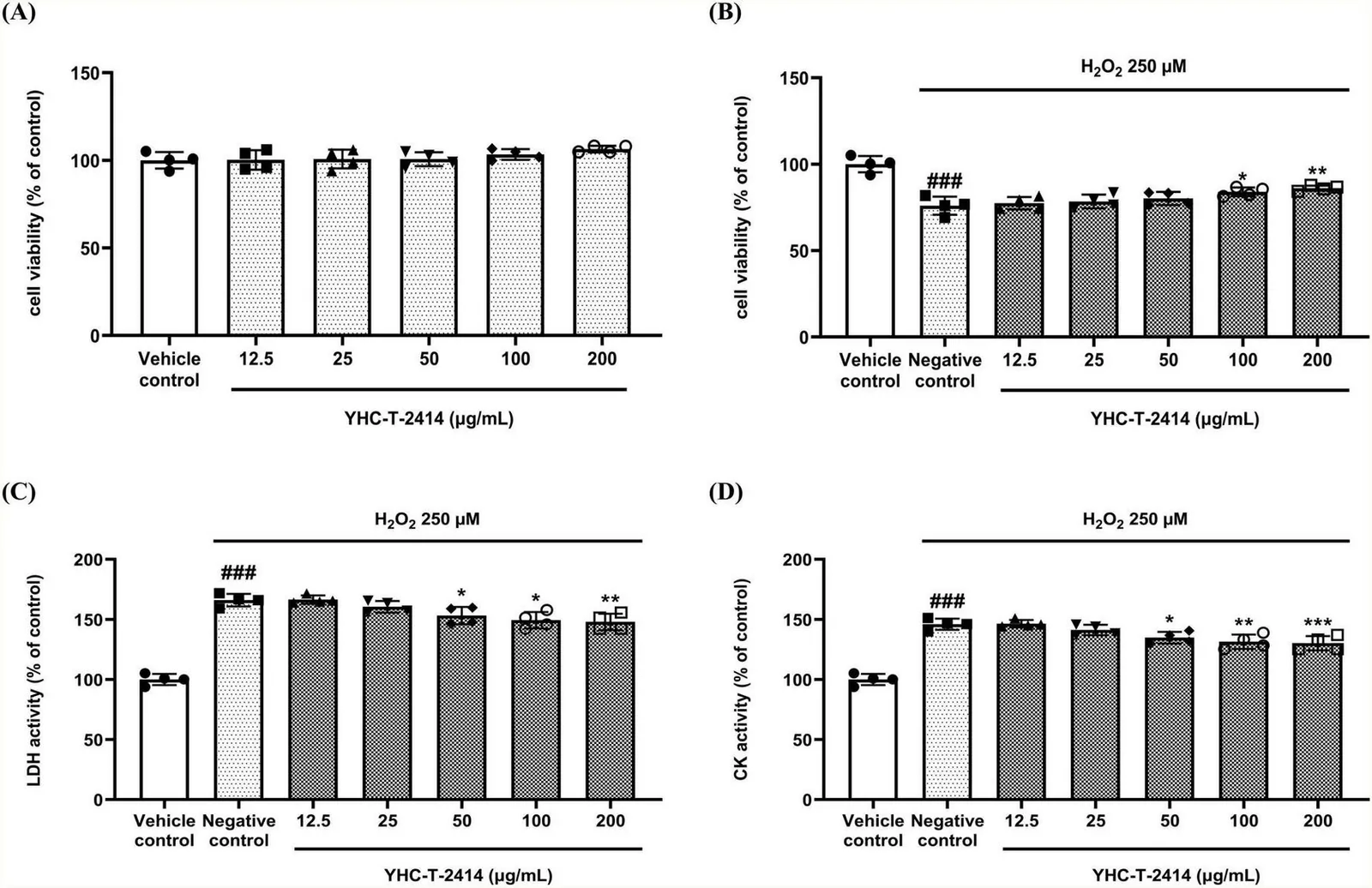
Effects of YHC-T-2414 on cell viability, LDH activity, and CK activity in normal and H_2_O_2_-treated C2C12 myoblast cells. **(A)** Cell viability in normal C2C12 cells treated with YHC-T-2414. **(B)** Cell viability in H_2_O_2_-treated C2C12 cells exposed to YHC-T-2414. **(C)** LDH and **(D)** CK activity in H_2_O_2_-treated C2C12 cells exposed to YHC-T-2414. H_2_O_2_, hydrogen peroxide. LDH, lactate dehydrogenase. CK, creatine kinase. Data are expressed as the mean ± standard deviation. ###*p* < 0.001 vs. vehicle control. *p < 0.05, **p < 0.01, ***p < 0.001 vs. negative control.

C2C12 myoblasts (ATCC, CRL-1772, Manassas, VA, United States) were maintained in Dulbecco’s Modified Eagle Medium (DMEM, Gibco, Thermo Fisher Scientific, Waltham, MA, United States) supplemented with 10% (v/v) fetal bovine serum (FBS, Gibco) and 1% (w/v) penicillin–streptomycin (P/S, Gibco). The cells were cultured at 37 °C in a humidified incubator containing 5% CO_2_. Cell viability was evaluated using an Ez-cytox assay (DoGenBio, Seoul, Republic of Korea). C2C12 cells (passages 8–12) were seeded in 96-well plates at a density of 1 × 10^4^ cells/well. YHC-T-2414 was initially dissolved in dimethyl sulfoxide (DMSO) to prepare a 1000x stock solution and subsequently diluted with culture medium to the desired working concentrations immediately before use. The final concentration of DMSO did not exceed 0.1% (v/v) in any treatment group. Vehicle control cells treated culture medium containing the corresponding concentration of DMSO (0.1%) without YHC-T-2414. Cells were treated with YHC-T-2414 at concentrations up to 200 μg/mL for 24 h. The maximum concentration (200 μg/mL) was selected because no cytotoxicity was observed in C2C12 myoblasts within this concentration range (Figure 2A). To induce oxidative damage, C2C12 cells were pretreated with YHC-T-2414 for 24 h, followed by incubation with 250 μM H_2_O_2_ for 3 h. Absorbance was measured at 450 nm using a SpectraMax microplate reader (Molecular Devices, San Jose, CA, United States). The results were expressed as a percentage relative to the vehicle control. All *in vitro* experiments were performed without induction of myogenic differentiation and were independently repeated at least three times.

### 2.5 LDH and CK activity in C2C12 myoblasts

C2C12 myoblasts (passages 8–12) were seeded in 96-well plates at a density of 1 × 10^4^ cells/well, pretreated with YHC-T-2414 for 24 h, and then exposed to 250 μM H_2_O_2_ for 3 h. The supernatant was collected, and the activities of lactate dehydrogenase (LDH) and creatine kinase (CK) were measured using an LDH assay kit (ab102526, Abcam, Waltham, MA, USA) and a CK assay kit (ab155901, Abcam), respectively. Briefly, 50 μL of cell supernatant was combined with an equal volume of reaction mixture and incubated at 37 °C for 15 min in the dark. Absorbance was measured at 450 nm using a SpectraMax microplate reader, and enzyme activity was calculated using the following equation: enzyme activity (milliunit/mL) = ΔNADH × dilution factor/(reaction time × volume). All experiments were independently performed at least three times.

### 2.6 DPPH and ABTS free radical scavenging activity

The free radical scavenging activities of YHC-T-2414 and ascorbic acid against 2,2-diphenyl-1-picrylhydrazyl (DPPH) and 2,2’-azino-bis (3-ethylbenzothiazoline-6-sulfonic acid) (ABTS) were evaluated according to a previously reported method (18). For the DPPH radical scavenging assay, 0.25 mL of each sample was added to 1.75 mL of 0.05 mM DPPH solution (DG-DPH400, DoGenBio) prepared in methanol (Sigma-Aldrich, St. Louis, MO, United States). The mixture was incubated in the dark for 30 min, and the absorbance was measured at 517 nm using a spectrophotometer (UV-1601, Shimadzu, Japan). For the ABTS radical scavenging assay, a solution was prepared by mixing 7 mM ABTS (Sigma-Aldrich) with 2.45 mM potassium persulfate (Sigma-Aldrich), and then diluted with ethanol to obtain an absorbance of 0.700 at 734 nm. Subsequently, 1.9 mL of this solution was combined with 50 μL of each sample and incubated in the dark for 6 min, after which absorbance was measured at 734 nm using a spectrophotometer (UV-1601, Shimadzu). The results were expressed as radical scavenging activity and calculated as the percentage of remaining DPPH or ABTS radicals (%) using the following equation: Remaining radical (%) = 100−(Abs of blank−Abs of the sample)/Abs of blank × 100.

### 2.7 ROS assay

To assess the protective effects of YHC-T-2414 against oxidative stress–induced damage, intracellular ROS levels were measured in C2C12 myoblasts using CM-H_2_DCFDA, a general oxidative stress indicator. Cells (passages 8–12) were seeded in 96-well plates at a density of 1 × 10^4^ cells/well and pretreated with YHC-T-2414 at concentrations up to 200 μg/mL for 24 h. Oxidative stress was then induced by exposure to 250 μM H_2_O_2_ for an additional 3 h. ROS levels were determined using a CM-H_2_DCFDA assay kit (C6827, Invitrogen, Carlsbad, CA, United States) following the manufacturer’s protocol. After removal of the culture medium, cells were washed with phosphate-buffered saline (PBS, Gibco) and incubated with 10 μM CM-H_2_DCFDA in serum-free DMEM for 1 h at 37°C in the dark under a humidified 5% CO_2_ atmosphere. Fluorescence intensity was measured using a fluorescence spectrophotometer (SpectraMax M2, Molecular Devices) at excitation and emission wavelengths of 492 and 517 nm, respectively, and expressed as a percentage relative to the vehicle control. All experiments were independently performed at least three times.

### 2.8 Western blotting

C2C12 myoblasts (passages 8–12) were seeded in 6-well plates at a density of 2 × 10^5^ cells/well and cultured for 24 h. The cells were pretreated with YHC-T-2414 (25, 50, 100, and 200 μg/mL) for 24 h, followed by exposure to 250 μM H_2_O_2_ for an additional 3 h to induce oxidative stress. Protein lysates (30 μg) were separated on 4%–20% SDS–polyacrylamide gels (Bio-Rad, Hercules, CA, USA) and transferred onto nitrocellulose membranes (Amersham, Cytiva, Marlborough, MA, USA). The membranes were blocked with 5% bovine serum albumin (BSA; MP Biomedicals, Santa Ana, CA, USA) for 1 h, and then incubated with primary antibodies overnight at 4 °C. After washing, the membranes were incubated with horseradish peroxidase (HRP)-conjugated secondary antibodies for 1 h at room temperature. Protein bands were detected using enhanced chemiluminescence (ECL; Nippon Genetics Europe, Düren, Germany) after washing with Tris-buffered saline containing 0.05% Tween 20 (Tech & Innovation, Chuncheon, Republic of Korea). The signals were visualized using a MicroChemi imaging system (DNR Bio-Imaging Systems, Neve Yamin, Israel). Band intensities were quantified using ImageJ software (National Institutes of Health, Bethesda, MD, United States) and normalized to GAPDH. All western blot experiments were independently conducted three times (*n* = 3).

### 2.9 Experimental animals

All experimental procedures were approved by the Institutional Animal Care and Use Committee and conducted in compliance with the “Animal Care Act” established by the Ministry of Agriculture and Forestry, Republic of Korea. Additional approval was obtained from the Animal Ethics Committee of Jeonbuk National University Hospital Non-Clinical Evaluation Center (Approval No. JBNUH-IACUC-2024-29). A total of 40, 6-week-old male ICR mice weighing 32.4 ± 1.4 g (Koatech, Pyeongtaek, Republic of Korea) were used in this study. The mice were maintained in a specific pathogen-free animal facility at Jeonbuk National University Hospital (Jeonju, Republic of Korea) and acclimated for at least 7 days prior to the study. Following acclimation, body weights were recorded, and animals were randomly assigned to groups (*n* = 10 per group) to ensure comparable mean body weights. The housing conditions were controlled as follows: temperature, 20 °C–26 °C; relative humidity, 40%–70%; ventilation rate, 12 times/h; light/dark cycle, lights on at 8:00 a.m. and off at 8:00 p.m.; and light intensity, 150–300 Lux. Animals had free access to water and a standard diet (AIN-76A, Orient Bio, Seongnam, Republic of Korea).

### 2.10 Experimental design and treatment

Animals were randomly allocated into four groups (*n* = 10 per group): a normal control (vehicle), a positive control (creatine, 500 mg/kg), and YHC-T-2414 treatment groups (12.5 and 25 mg/kg/day). The normal control group received olive oil, which served as the solvent for YHC-T-2414. YHC-T-2414 was administered once daily for 21 days at 12.5 or 25 mg/kg, while creatine (Sigma-Aldrich) served as a positive control at 500 mg/kg. The selected doses of YHC-T-2414 were based on the recommended daily intake of astaxanthin (4–12 mg) specified in the Korean Health Functional Food Code issued by the Ministry of Food and Drug Safety (Republic of Korea). Since YHC-T-2414 contains 100 mg of astaxanthin per gram of extract, the highest recommended daily intake of astaxanthin (12 mg) corresponds to 120 mg/day of YHC-T-2414. This human dose was converted to the equivalent mouse dose using the body surface area normalization method, yielding approximately 25 mg/kg. An additional lower dose (12.5 mg/kg), corresponding to one-half of the calculated dose, was included to evaluate dose-dependent effects. Furthermore, these doses were substantially lower than those reported to be safe in previous toxicological studies of astaxanthin derived from *H. pluvialis*, further supporting the appropriateness of the dose selection (19). On day 21, mice were anesthetized and sacrificed following administration of ketamine and rompun. Blood samples were collected via the abdominal aorta into EDTA-2K tubes (BD Vacutainer, Becton Dickinson, Franklin Lakes, NJ, United States) and centrifuged at 3,000 rpm for 10 min to separate the serum. The serum was stored at −80 °C until further analysis. After skin removal, soleus muscles were dissected from both hind limbs, rapidly frozen in liquid nitrogen, and stored at −80 °C until use.

### 2.11 Weight-loaded forced swimming test

A weight-loaded forced swimming test (FST) was performed to assess the anti-fatigue effects of YHC-T-2414. Following 1 week of acclimatization, mice were randomly assigned to the experimental groups (*n* = 10 per group). Mice were individually placed in a transparent cylindrical acrylic tank (30 cm in diameter) containing distilled water at a depth of 30 cm, which prevented their tails from touching the bottom. The water temperature was maintained at 22 °C ± 1 °C throughout the experiment. To increase the workload, a weight corresponding to 5% of each mouse’s body weight was attached. The investigator responsible for recording the swimming time was blinded to the treatment allocation throughout the experiment. The time to exhaustion was recorded, with exhaustion defined as the point at which the mouse failed to rise to the surface for breathing and remained submerged for at least 7 s. To allow adaptation to the swimming conditions, two training sessions (approximately 30 min each) were conducted on days 14 and 18 of the treatment period. The final FST was carried out on day 21, 1 h after administration of YHC-T-2414.

### 2.12 Biochemistry

Blood samples were collected and centrifuged at 3,000 rpm for 10 min to obtain serum. The serum was subsequently used to analyze levels of LDH, CK, lactate, MDA, tumor necrosis factor-α (TNF-α), and interleukin-6 (IL-6) using commercially available assay kits. Specifically, LDH (ab102526) and CK (ab155901) kits were purchased from Abcam, lactate (MA-LAC) and MDA (MA-MDA-2) kits from RayBiotech (Peachtree Corners, GA, United States), and ELISA kits for TNF-α (BMS607-3) and IL-6 (BMS603HS) from Invitrogen. All procedures were performed in accordance with the manufacturers’ protocols.

### 2.13 LDH in muscle

Soleus muscle tissues were collected, placed in tubes, and stored at −80 °C until analysis. For LDH measurement, approximately 10 mg of muscle tissue was homogenized in 100 μL of assay buffer on ice using the buffer supplied with the assay kit. The homogenates were centrifuged at 10,000 × *g* for 15 min, and the resulting supernatant was collected for further analysis. LDH activity in the muscle extracts was subsequently quantified using an LDH assay kit (ab102526, Abcam).

### 2.14 Statistical analysis

All *in vitro* experiments were performed in triplicate and independently repeated at least three times. Statistical analysis was conducted using one-way analysis of variance (ANOVA), followed by Dunnett’s multiple comparison test as a *post hoc* analysis. For *in vivo* data, group differences were also evaluated using one-way ANOVA with Dunnett’s *post-hoc* test. Results are presented as mean ± standard deviation (SD) and statistical significance was defined as *p* < 0.05. All statistical analyses were performed using GraphPad Prism software (version 9.0; GraphPad Software Inc., La Jolla, CA, United States).

## 3 Results

### 3.1 Effect of YHC-T-2414 on cell viability and H_2_O_2_-induced cytotoxicity in C2C12 myoblasts

The effects of YHC-T-2414 on C2C12 myoblasts’ viability and H_2_O_2_-induced cytotoxicity are shown in Figures 2A, B, respectively. Treatment with YHC-T-2414 up to 200 μg/mL showed a tendency to increase cell viability, although this effect was not statistically significant (Figure 2A). H_2_O_2_-induced cytotoxicity was dose-dependently attenuated by YHC-T-2414 treatment, with partial recovery of cell viability observed at concentrations of 100 and 200 μg/mL, showing increases of 10.53% and 13.32%, respectively (Figure 2B; *p < 0.05 and **p < 0.01). These results suggest that YHC-T-2414 at concentrations up to 200 μg/mL does not affect the viability of C2C12 myoblasts under normal conditions, but may alleviate H_2_O_2_-induced cytotoxicity.

### 3.2 Effect of YHC-T-2414 on LDH and CK activities in C2C12 myoblasts

The effects of YHC-T-2414 on LDH and CK activities are shown in Figures 2C,D, respectively. Treatment with 250 μM H_2_O_2_ significantly increased LDH activity by 66.00% compared with the vehicle control (###*p* < 0.001). In contrast, LDH activity was gradually decreased with increasing concentrations of YHC-T-2414, with a significant reduction of up to 10.89% at 200 μg/mL (Figure 2C; **p < 0.01). Similarly, exposure to 250 μM H_2_O_2_ significantly increased CK activity by 46.03% compared with the vehicle control (###*p* < 0.001). In contrast, CK activity was gradually reduced with increasing concentrations of YHC-T-2414, with a significant inhibitory effect observed at 200 μg/mL, where CK activity was reduced by 10.85% (Figure 2D; ***p < 0.001). These findings indicate that YHC-T-2414 may attenuate H_2_O_2_-induced increases in LDH and CK activities.

### 3.3 Effect of YHC-T-2414 on DPPH and ABTS radical scavenging activities and intracellular ROS levels

**FIGURE 3:**
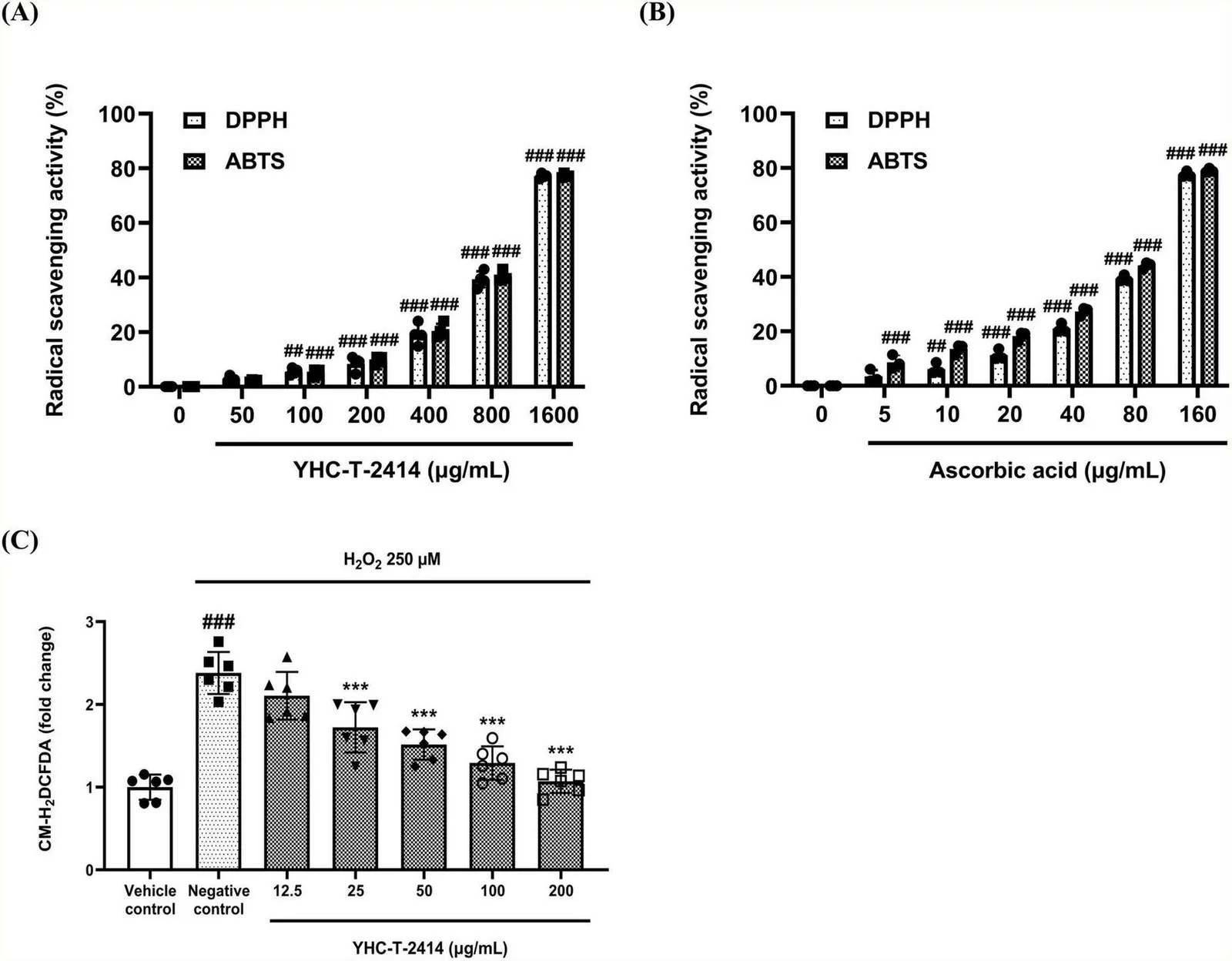
Effects of YHC-T-2414 on DPPH and ABTS radical scavenging activity and CM-H_2_DCFDA production in normal and H_2_O_2_-treated C2C12 myoblast cells. DPPH and ABTS radical scavenging activity (%) following **(A)** YHC-T-2414 and **(B)** ascorbic acid treatments. **(C)** Measurement of CM-H_2_DCFDA production, a ROS indicator, in H_2_O_2_-treated C2C12 cells exposed to YHC-T-2414. H_2_O_2_, hydrogen peroxide; DPPH, 2,2-diphenyl-1-picrylhydrazyl; ABTS, 2,2’-azino-bis (3-ethyl-benzothiazoline-6-sulfonic acid); ROS, reactive oxygen species; CM-H_2_DCFDA: 5-(and-6)-chloromethyl-2’,7’-dichlorodihydrofluorescein diacetate. Data are expressed as the mean ± standard deviation. ##*p* < 0.01, ###*p* < 0.001 vs. vehicle control. ***p < 0.001 vs. negative control.

The results of the DPPH and ABTS assays performed to assess the *in vitro* antioxidant effects of YHC-T-2414, as well as its effects on intracellular ROS levels, are presented in Figures 3A–C. YHC-T-2414 was tested at concentrations up to 1,600 μg/mL for the DPPH and ABTS assays, and showed dose-dependent increase in radical scavenging activities was observed from 100 to 1,600 μg/mL (Figure 3A; ##*p* < 0.01, ###*p* < 0.001). Ascorbic acid, used as a positive control, was tested at concentrations 10-fold lower (5–160 μg/mL) than YHC-T-2414 (Figure 3B). The DPPH radical scavenging activity of ascorbic acid increased significantly from 10 to 160 μg/mL (##*p* < 0.01, ###*p* < 0.001), while its ABTS radical scavenging activity increased significantly from 5 to 160 μg/mL (###*p* < 0.001). The effect of YHC-T-2414 on the inhibition of oxidative stress-induced damage in C2C12 myoblasts is shown in Figure 3C. Intracellular ROS levels were assessed using the CM-H_2_DCFDA assay (20). Treatment of C2C12 myoblasts with 250 μM H_2_O_2_ significantly increased ROS levels by 2.38-fold (###*p* < 0.001). YHC-T-2414 significantly reduced ROS levels from 25 μg/mL, with a notable 55.02% decrease observed at 200 μg/mL (***p < 0.001). These results suggest that YHC-T-2414 possesses antioxidant activity and may attenuate H_2_O_2_-induced intracellular ROS accumulation in C2C12 myoblasts.

### 3.4 Effects of YHC-T-2414 on the antioxidant (AMPK-NRF2-KEAP1) and mitochondrial biogenesis (AMPK-SIRT1-PGC-1α) pathways in C2C12 myoblasts

**FIGURE 4:**
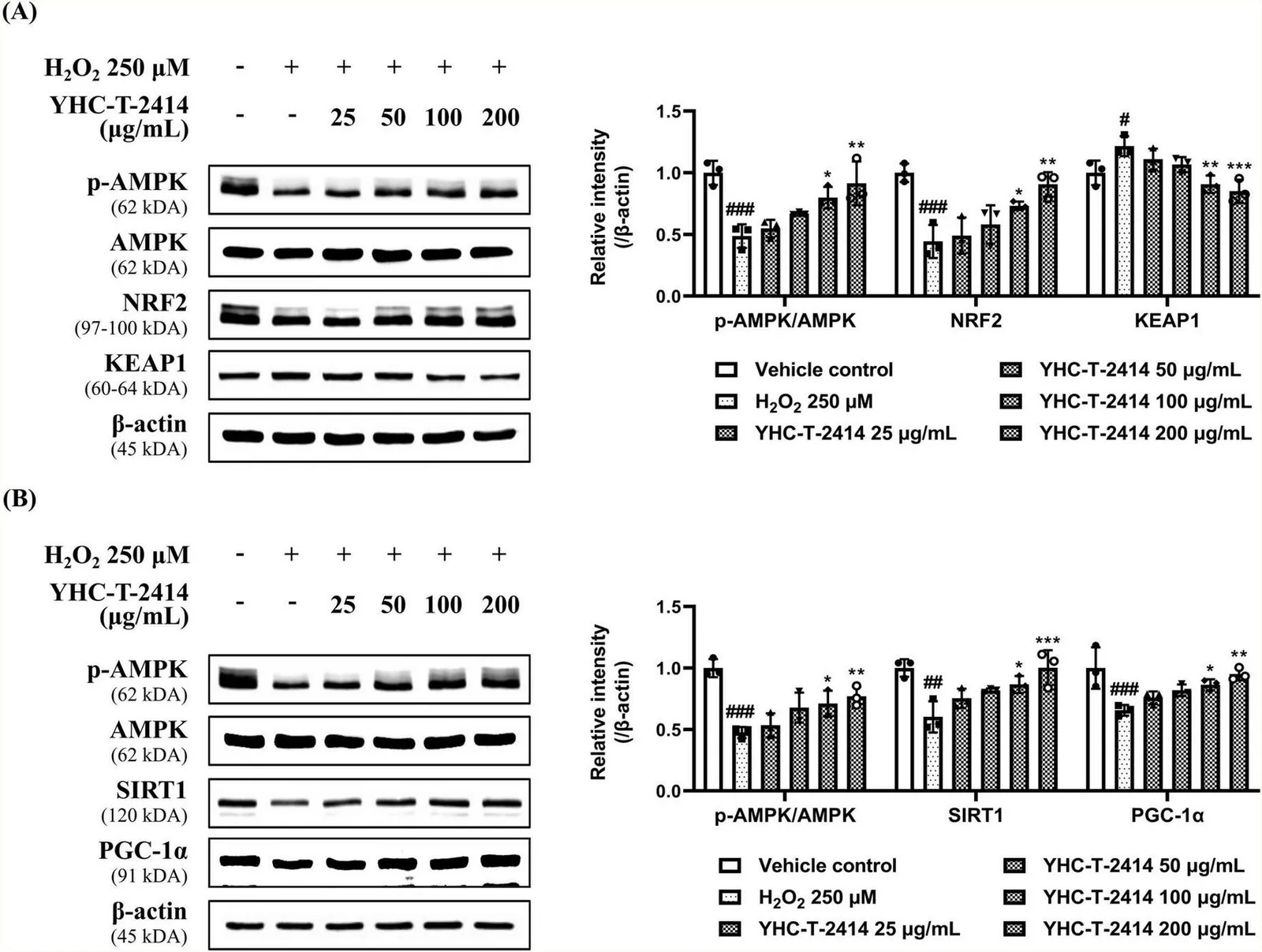
Effects of YHC-T-2414 on anti-fatigue-related mechanism in H_2_O_2_-treated C2C12 myoblast cells. Western blot analysis to investigate **(A)** antioxidant (AMPK–NRF2–KEAP1) and **(B)** mitochondrial biogenesis (AMPK–SIRT1–PGC-1α) mechanisms in H_2_O_2_-treated C2C12 cells exposed to YHC-T-2414. H_2_O_2_, hydrogen peroxide; AMPK, AMP-activated protein kinase; p-AMPK, phospho-AMPK; NRF2, nuclear factor erythroid 2-related factor 2; KEAP1, kelch-like ECH-associated protein 1; SIRT1, sirtuin 1; PGC-1α, peroxisome proliferator-activated receptor gamma coactivator 1-alpha. Data are expressed as the mean ± standard deviation (*n* = 3). #*p* < 0.05, ##*p* < 0.01, ###*p* < 0.001 vs. vehicle control. *p < 0.05, **p < 0.01, ***p < 0.001 vs. negative control.

The effects of YHC-T-2414 on the expression of proteins related to antioxidant activity (AMPK, NRF2, and KEAP1) and mitochondrial biogenesis (AMPK, SIRT1, and PGC-1α) are shown in Figure 4. In C2C12 cells subjected to H_2_O_2_-induced oxidative stress, the protein expression levels of phospho-AMPK (p-AMPK) and NRF2 were significantly decreased by 51.38% and 55.62% (###*p* < 0.001), respectively, whereas KEAP1 protein expression was significantly increased by 1.22-fold (#*p* < 0.05). Treatment with YHC-T-2414 (100 and 200 μg/mL) significantly increased the protein expression of p-AMPK by 1.64-fold (*p < 0.05) and 1.88-fold (**p < 0.01), respectively, and NRF2 expression by 1.65-fold (*p < 0.05) and 2.04-fold (**p < 0.01), respectively. In contrast, KEAP1 protein expression was significantly decreased by 25.41% (**p < 0.01) and 30.33% (***p < 0.001), respectively (Figure 4A). In H_2_O_2_-treated C2C12 cells, the protein expression levels of p-AMPK, SIRT1, and PGC-1α were significantly decreased by 52.47% (###*p* < 0.001), 39.66% (##*p* < 0.01), and 34.37% (###*p* < 0.001), respectively. Treatment with YHC-T-2414 (100 and 200 μg/mL) significantly increased the protein expression of p-AMPK by 1.49-fold (*p < 0.05) and 1.62-fold (**p < 0.01), SIRT1 by 1.44-fold (*p < 0.05) and 1.66-fold (***p < 0.001), and PGC-1α by 1.32-fold (*p < 0.05) and 1.56-fold (**p < 0.01), respectively (Figure 4B). These findings suggest that YHC-T-2414 may exert antioxidant effects and promote mitochondrial biogenesis through modulation of the AMPK-NRF2-KEAP1 and AMPK-SIRT1-PGC-1α signaling pathways, thereby potentially contributing to fatigue alleviation.

### 3.5 Effect of YHC-T-2414 on swimming duration in the FST

**FIGURE 5:**
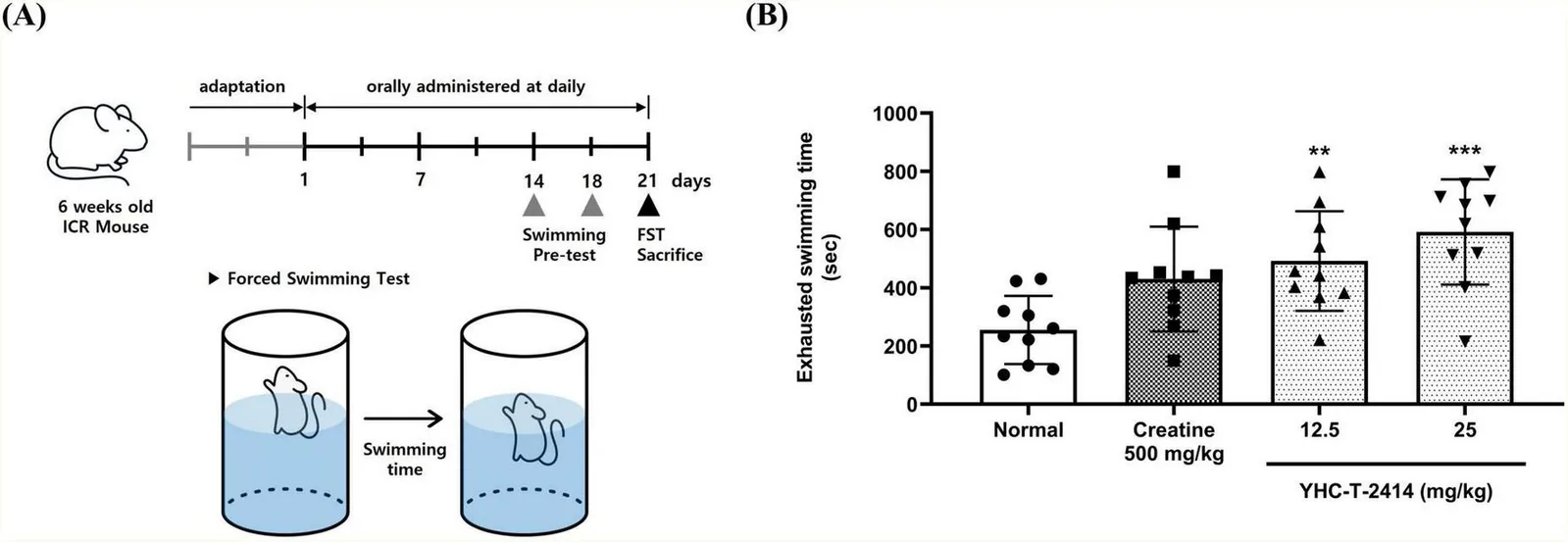
Experimental design and effects of YHC-T-2414 on FST in mice. Six-week-old male ICR mice were acclimated for 1 week and then orally administered YHC-T-2414 (12.5 and 25 mg/kg) or 500 mg/kg creatine for 21 consecutive days. The experimental schedule included a swimming pre-test on day 14 and 18, followed by the FST and sacrifice on day 21. **(A)** Schematic representation of the experimental timeline and FST procedure. **(B)** Exhaustive swimming time during the FST to assess physical endurance. Creatine was used as the positive control. FST, forced swimming test. Data are expressed as mean ± standard deviation (*n* = 10 per group). **p < 0.01, ***p < 0.001 vs. normal control.

The scheme of the animal study based on the FST and the effect of YHC-T-2414 on swimming duration are shown in Figures 5A,B, respectively. For the normal control mice, swimming continued for 255.10 s in the FST following the 3-week treatment with YHC-T-2414 (Figure 5B). In the positive control group administered creatine (500 mg/kg), the exhausted swimming time was 430.30 s, representing a 68.68% increase compared with the normal control group; however, this increase was not statistically significant. In contrast, administration of YHC-T-2414 (12.5 and 25 mg/kg) resulted in a significant increase in exhausted swimming time by 92.90% (**p < 0.01) and 131.95% (***p < 0.001), respectively, compared with the normal control group. Taken together, these findings suggest that YHC-T-2414 administration prolonged exhausted swimming time, indicating its potential to improve exercise endurance.

### 3.6 Effects of YHC-T-2414 on fatigue-related blood biochemical parameters and muscle metabolites in mice after the FST

**FIGURE 6:**
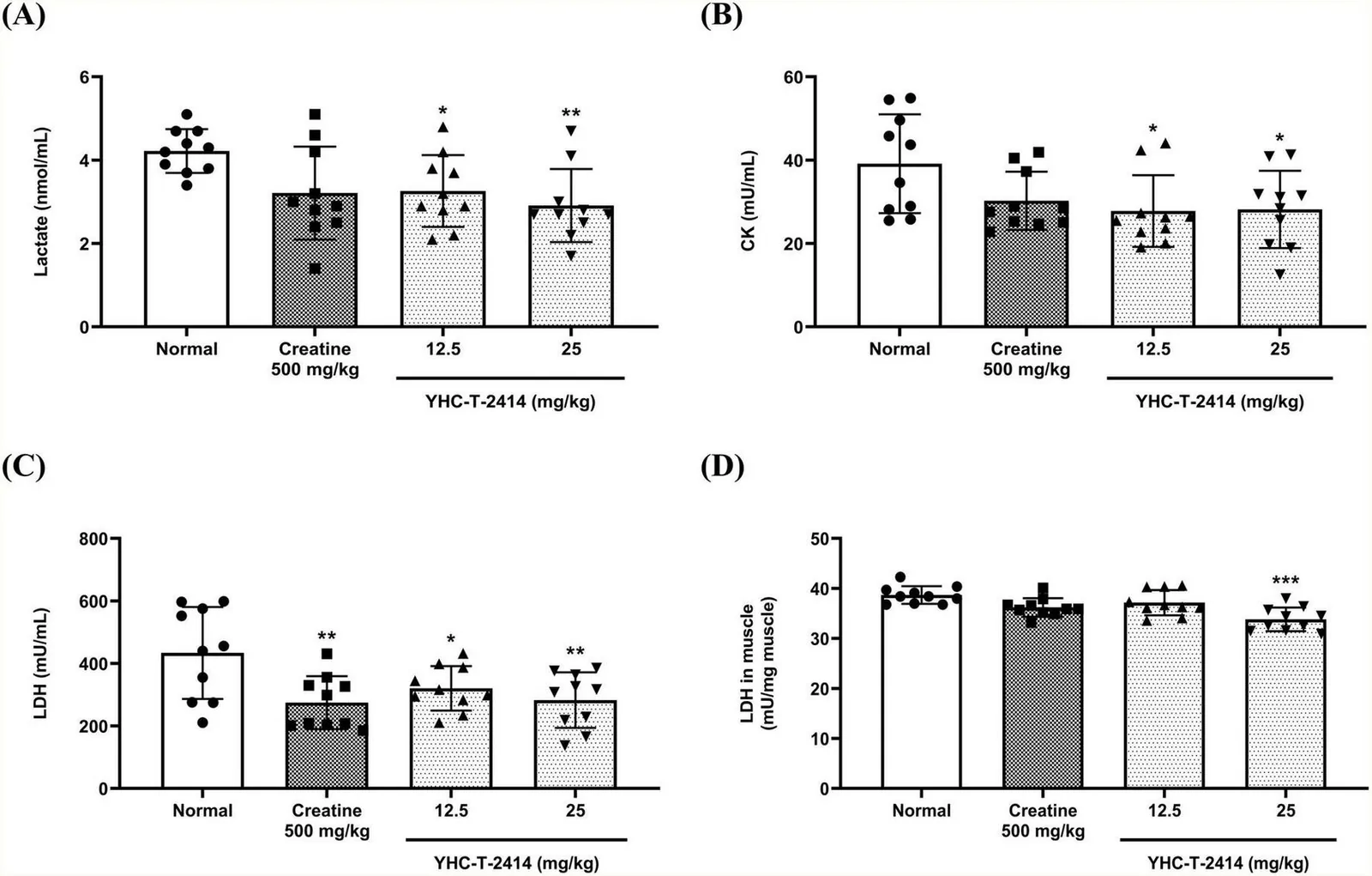
Effects of YHC-T-2414 on blood biochemical parameters and muscle metabolites in mice after FST. Mice orally administered YHC-T-2414 (12.5 and 25 mg/kg) or 500 mg/kg creatine for 21 consecutive days. The experimental schedule included a swimming pre-test on day 14 and 18, followed by the FST and sacrifice on day 21. After sacrifice, serum and soleus muscle were collected and used for biomarker analysis. Levels of **(A)** lactate, **(B)** CK, and **(C)** LDH were evaluated in the serum, and **(D)** LDH levels in muscle were measured in the soleus muscle. Creatine was used as the positive control. FST, forced swimming test; CK, creatine kinase; LDH, lactate dehydrogenase. Data are expressed as mean ± standard deviation (*n* = 10 per group). *p < 0.05, **p < 0.01, ***p < 0.001 vs. normal control.

The effects of YHC-T-2414 on fatigue-related blood biochemical parameters (lactate, CK, and LDH) and muscle LDH levels in mice after the FST are presented in Figure 6. Compared with the normal control group, which exhibited a blood lactate level of 4.22 nmol/mL, the positive control group showed a significantly reduced level of 3.20 nmol/mL, corresponding to a 24.17% decrease (*p < 0.05). In the YHC-T-2414-treated groups (12.5 and 25 mg/kg), blood lactate levels were 3.26 and 2.90 nmol/mL, representing reductions of 22.75% (*p < 0.05) and 31.28% (**p < 0.01), respectively, compared with the control group (Figure 6A).

For blood CK levels, the positive control group exhibited a 22.73% reduction (30.26 mU/mL) compared with the normal control group (39.16 mU/mL), with no statistically significant difference. Blood CK levels in the YHC-T-2414-treated groups at 12.5 and 25 mg/kg were 27.79 and 28.19 mU/mL, respectively, corresponding to significant reductions of 29.05% and 28.03% compared with the normal control group (Figure 6B; *p < 0.05).

For blood LDH levels, the positive control group showed a significant 36.60% reduction (274.91 mU/mL) compared with the normal control group (433.60 mU/mL) (**p < 0.01). Likewise, blood LDH levels in the YHC-T-2414-treated groups at 12.5 and 25 mg/kg were significantly decreased by 26.12% (320.35 mU/mL) (*p < 0.05) and 34.79% (282.74 mU/mL) (**p < 0.01) relative to the normal control group (Figure 6C). LDH levels in muscle were significantly reduced by 6.33% in the positive control group (36.23 mU/mg) compared with the normal control group (38.68 mU/mg) (Figure 6D; *p < 0.05), and by 12.56% in the group administered 25 mg/kg YHC-T-2414 (33.82 mU/mg) relative to the normal control group (Figure 6D; ***p < 0.001). These findings suggest that YHC-T-2414 may exhibit anti-fatigue effects, as evidenced by the reduced levels of fatigue-related blood biomarkers and muscle LDH following the FST.

### 3.7 Effect of YHC-T-2414 on antioxidant- and anti-inflammatory-related blood biochemical parameters in mice after the FST

**FIGURE 7:**
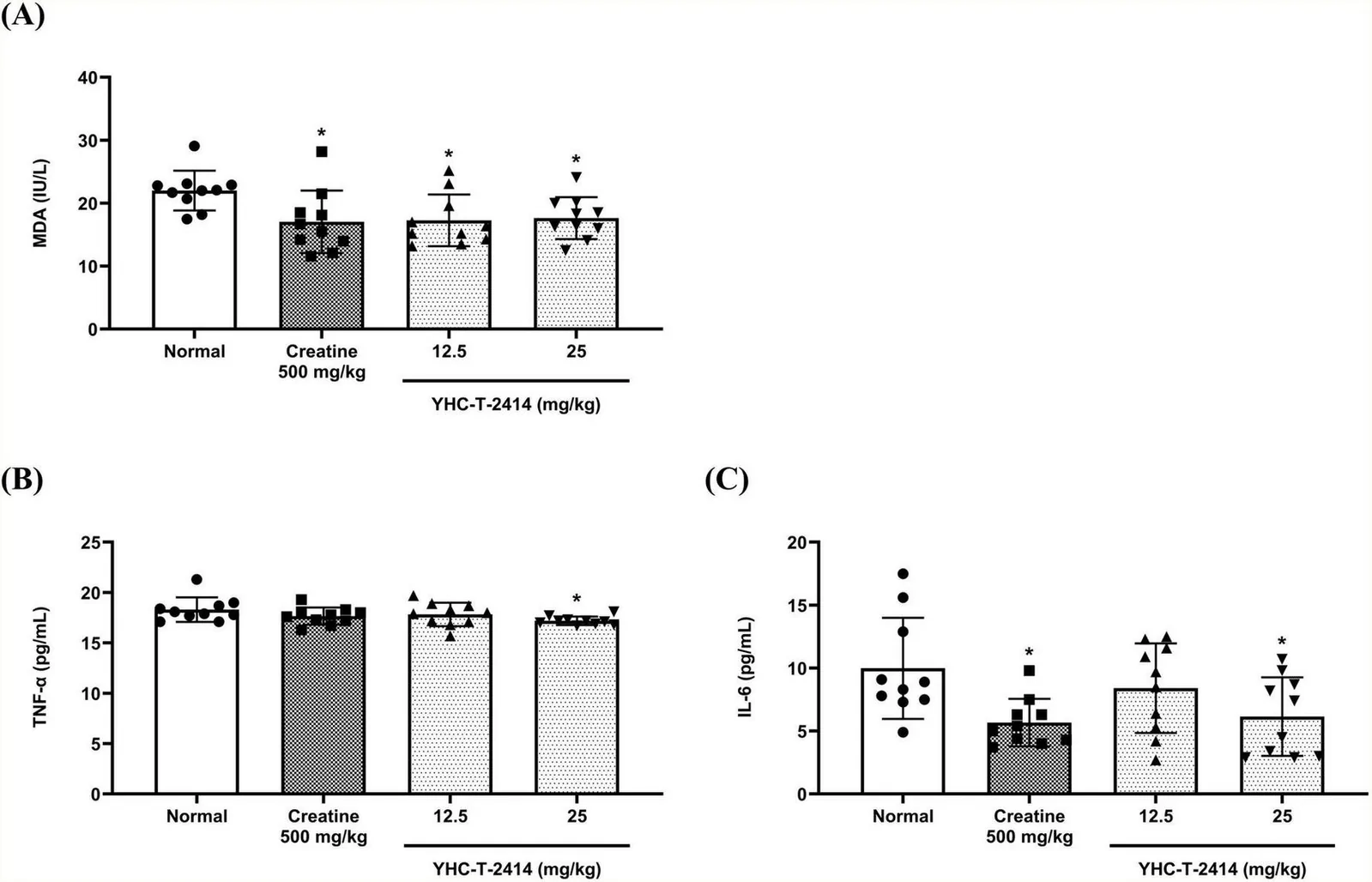
Effects of YHC-T-2414 on oxidative stress and inflammatory markers in mouse serum after FST. Mice orally administered YHC-T-2414 (12.5 and 25 mg/kg) or 500 mg/kg creatine for 21 consecutive days. The experimental schedule included a swimming pre-test on day 14 and 18, followed by the FST and sacrifice on day 21. After sacrifice, serum was collected and used for biomarker analysis. Levels of **(A)** the lipid peroxidation marker MDA and pro-inflammatory cytokines including **(B)** TNF-α and **(C)** IL-6 were measured in serum. Creatine was used as the positive control. MDA, malondialdehyde; TNF-α, tumor necrosis factor-alpha; IL-6, interleukin-6. Data are expressed as mean ± standard deviation (*n* = 10 per group). *p < 0.05 vs. normal control.

The effects of YHC-T-2414 on blood MDA levels, an indicator of oxidative stress, and on the inflammatory biomarkers TNF-α and IL-6 in mice subjected to the FST are presented in Figure 7. The blood MDA level in the normal control group was 22.01 IU/L, whereas the positive control group exhibited a significantly reduced blood MDA level of 17.04 IU/L, corresponding to a 22.58% decrease. Similarly, in the groups administered YHC-T-2414 at two doses (12.5 and 25 mg/kg), blood MDA levels were 17.27 and 17.63 IU/L, representing significant reductions of 21.54% and 19.90%, respectively, relative to the normal control group (Figure 7A; *p < 0.05).

Blood TNF-α levels were decreased by 3.50% in the positive control group (17.65 pg/mL) and by 2.52% in the 12.5 mg/kg YHC-T-2414 group (17.83 pg/mL) compared with the normal control group (18.29 pg/mL); however, these reductions were not statistically significant. In contrast, blood TNF-α levels in the group administered 25 mg/kg YHC-T-2414 (17.18 pg/mL) were significantly reduced by 6.07% compared with the normal control group (Figure 7B; *p < 0.05). Blood IL-6 levels were significantly reduced by 43.13% in the positive control group (5.67 pg/mL) (*p < 0.05) and by 38.42% in the 25 mg/kg YHC-T-2414 group (6.14 pg/mL) (*p < 0.05) compared with the normal control group (9.97 pg/mL). In contrast, blood IL-6 levels in the 12.5 mg/kg YHC-T-2414 group (8.40 pg/mL) were decreased by 15.75% relative to the normal control group, though this reduction was not statistically significant (Figure 7C). These results indicate that administration of YHC-T-2414 may reduce oxidative stress and inflammatory biomarkers in mice subjected to the FST, thereby potentially contributing to fatigue alleviation.

## 4 Discussion

To evaluate the anti-fatigue effects of YHC-T-2414 prior to conducting the FST in mice, an *in vitro* study using C2C12 myoblasts was performed. Treatment with YHC-T-2414 did not affect cell viability in normal C2C12 myoblasts (Figure 2A). Moreover, YHC-T-2414 significantly and dose-dependently attenuated cell viability in H_2_O_2_-treated C2C12 myoblast injury models (Figure 2B). Murine-derived C2C12 myoblasts are widely used as an *in vitro* model for evaluating anti-fatigue efficacy based on the assessment of fatigue-related biomarkers, such as LDH and CK activities, as well as for exploring underlying molecular mechanisms (21, 22). H_2_O_2_, a representative form of ROS, is commonly used to induce intracellular oxidative stress, thereby reproducing fatigue-related pathological conditions such as muscle cell damage, mitochondrial dysfunction, and ROS accumulation (23, 24). LDH and CK activities were evaluated to determine the anti-fatigue effects of YHC-T-2414 in H_2_O_2_-treated C2C12 myoblasts. LDH is an enzyme widely distributed in most tissues and catalyzes the interconversion of pyruvate and lactate (25). Lactate is a well-known indicator of muscle fatigue. It accumulates in the body when a glycolytic imbalance occurs due to repeated muscle contractions during high-intensity exercise (26). CK facilitates the reversible transfer of a phosphate group between creatine and ATP to form phosphocreatine, which serves as a rapid energy reserve. However, CK is released into the circulation when muscle fibers are damaged (27). Therefore, increases in LDH and CK activities are considered indicative of muscle damage and have been widely used as representative biomarkers in various anti-fatigue studies. In the present study, H_2_O_2_ treatment significantly increased LDH and CK activities, whereas YHC-T-2414 treatment significantly and dose-dependently reduced LDH and CK activities starting at 50 μg/mL (Figures 2C,D). Collectively, the present results indicate that YHC-T-2414 was non-cytotoxic at concentrations up to 200 μg/mL, mitigated H_2_O_2_-induced reductions in C2C12 cell viability, and attenuated the elevation of LDH and CK activities under oxidative stress conditions. Accordingly, these results suggest that YHC-T-2414 may protect C2C12 myoblasts against H_2_O_2_-induced oxidative damage and support its potential anti-fatigue effects.

Physical fatigue is defined as a state in which the body fails to recover after repetitive or excessive exercise and is unable to maintain normal levels of physical activity (28). Although the exact mechanisms remain unclear, an imbalance in the endogenous antioxidant system induced by high-intensity exercise is considered one of the most representative causes (29). Excessive energy expenditure during strenuous exercise increases ROS production, which damages mitochondria and cell membranes and induces intracellular metabolic disturbances, ultimately leading to fatigue (30). The alleviation of oxidative stress is considered a key factor in improving fatigue. Therefore, the antioxidant capacity of YHC-T-2414 was evaluated by measuring DPPH and ABTS radical scavenging activities, as well as CM-H_2_DCFDA, a general indicator of oxidative stress. YHC-T-2414 showed free radical scavenging activity against DPPH and ABTS, although at concentrations approximately 10-fold higher than those of the positive control, ascorbic acid (Figures 3A,B). Furthermore, in H_2_O_2_-induced oxidative stress conditions in C2C12 myoblasts, YHC-T-2414 significantly and dose-dependently reduced CM-H_2_DCFDA levels starting at 25 μg/mL (Figure 3C). These findings suggest that the antioxidant activity of YHC-T-2414 may contribute to its potential anti-fatigue effects by attenuating oxidative stress.

To investigate the mechanism underlying the antioxidant activity of YHC-T-2414, the activation of the AMPK–NRF2–KEAP1 signaling pathway was analyzed. NRF2 is a key transcription factor involved in cellular responses to oxidative stress, regulating antioxidant and detoxification pathways. Under basal conditions, NRF2 is sequestered in the cytoplasm through binding to KEAP1 and is targeted for degradation. However, under oxidative stress, NRF2 dissociates from KEAP1 and translocates into the nucleus, where it induces the expression of antioxidant enzymes (31). AMPK is an enzyme that responds to the intracellular energy status and is activated when ATP levels are depleted, thereby playing a critical role in maintaining cellular energy homeostasis (9, 32). Previous studies have also shown that phosphorylated AMPK promotes NRF2 release from KEAP1 and enhances its nuclear translocation (33). Based on these findings, we examined whether YHC-T-2414 activates the AMPK–NRF2–KEAP1 signaling pathway in C2C12 myoblasts. In C2C12 myoblasts exposed to H_2_O_2_-induced oxidative stress, the protein expression levels of p-AMPK and NRF2 were markedly reduced. In contrast, treatment with YHC-T-2414 at 100 and 200 μg/mL increased the expression of these proteins to levels comparable to the vehicle control. Conversely, KEAP1 protein expression, which was elevated by H_2_O_2_ exposure, was significantly downregulated in the YHC-T-2414-treated groups at 100 and 200 μg/mL (Figure 4A). These findings suggest that YHC-T-2414 may modulate the AMPK–NRF2–KEAP1 signaling pathway. However, because NRF2 protein expression was evaluated using whole-cell lysates, the present study did not directly evaluate NRF2 nuclear translocation. Further evaluation of NRF2 nuclear localization and downstream antioxidant target genes would strengthen the mechanistic evidence for NRF2 pathway activation by YHC-T-2414.

Recently, exercise-induced mitochondrial biogenesis has been recognized as a key adaptive mechanism contributing to improved endurance performance and resistance to fatigue (34). This process enhances mitochondrial content, oxidative capacity, and energy metabolism, and is primarily regulated by signaling pathways involving AMPK and PGC-1α (35). As the primary organelles responsible for most of the intracellular ATP production, mitochondria play a central role in cellular energy metabolism. Accordingly, impaired mitochondrial function results in decreased ATP generation, which reduces muscle contractile capacity and ultimately contributes to fatigue development (12). SIRT1 is an enzyme involved in deacetylation and is known to activate PGC-1α by deacetylation (36). PGC-1α is widely regarded as a key transcriptional coactivator that plays a central role in regulating mitochondrial biogenesis and oxidative metabolism (37). Previous studies have shown that phosphorylation of AMPK activates SIRT1 and PGC-1α by increasing their protein expression levels. Activated PGC-1α subsequently translocates into the nucleus, where it directly regulates mitochondrial biogenesis (35). Furthermore, increased mitochondrial content mediated by PGC-1α can suppress ROS generation through intrinsic mitochondrial functions, thereby linking mitochondrial biogenesis to antioxidant activity (37). Based on these findings, we further examined whether YHC-T-2414 modulates the AMPK–SIRT1–PGC-1α signaling pathway, which is associated with mitochondrial biogenesis. In C2C12 myoblasts subjected to H_2_O_2_-induced oxidative stress, the protein expression levels of p-AMPK, SIRT1, and PGC-1α were significantly suppressed. However, treatment with YHC-T-2414 significantly increased the expression of these proteins to levels comparable to those of the vehicle control starting at a concentration of 100 μg/mL (Figure 4B). Collectively, these findings suggest that the potential anti-fatigue effects of YHC-T-2414 may be associated with modulation of the AMPK-NRF2-KEAP1 pathway involved in antioxidant defense and the AMPK-SIRT1-PGC-1α pathway associated with mitochondrial biogenesis. It should be noted that these molecular mechanisms were evaluated only in C2C12 myoblasts and were not validated in skeletal muscle tissues from the animal model. Therefore, further studies are needed to confirm whether these signaling pathways are similarly regulated *in vivo* following YHC-T-2414 administration.

In line with the *in vitro* results, an FST was performed in mice to assess the anti-fatigue effects of YHC-T-2414 (Figure 5A). Although the concentrations used in the *in vitro* experiments cannot be directly compared with the oral doses administered *in vivo* because orally administered compounds undergo absorption, distribution, metabolism, and excretion, the *in vitro* experiments were designed to investigate the underlying cellular mechanisms, whereas the *in vivo* study was conducted to evaluate the overall physiological anti-fatigue efficacy following oral administration. The FST is widely used for evaluating anti-fatigue activity in rodents and has been well established as a reliable experimental model in previous studies (38). Creatine promotes ATP supply required for exercise by regulating CK activity and the phosphocreatine system (39). Since creatine has been widely used as a positive control in previous studies investigating anti-fatigue effects, it was similarly used in the present study (40). Oral administration of YHC-T-2414 at doses of 12.5 and 25 mg/kg for 21 days significantly prolonged the exhaustion swimming time compared with the normal control group. Moreover, exhaustion swimming time was also significantly increased in the YHC-T-2414-treated groups compared with the positive control group (Figure 5B). Collectively, these findings suggest that YHC-T-2414 may exert anti-fatigue effects, as evidenced by the prolonged exhaustion swimming time in the FST.

To evaluate the effects of YHC-T-2414 on fatigue-related biomarkers, several biochemical parameters, including serum lactate, CK, and LDH, as well as LDH levels in muscle tissue, were assessed. Serum levels of lactate, CK, and LDH were reduced in all YHC-T-2414-treated groups compared with the normal control group, with effects comparable to or greater than those observed in the creatine-treated positive control group (Figures 6A–C). Muscle LDH also showed a decreasing trend in the YHC-T-2414-treated groups; however, no statistically significant difference was detected in the 12.5 mg/kg group. This finding may be explained by the rapid leakage of LDH from muscle into the bloodstream rather than its prolonged retention in the tissue (41), as well as by the abundant presence of LDH in tissues, which results in relatively small changes in muscle LDH levels (25). Therefore, muscle LDH levels should be interpreted together with serum LDH results (Figure 6D). Taken together, these findings suggest that YHC-T-2414 may reduce fatigue-related biochemical markers, reflecting a potential protective effect against exercise-induced muscle damage.

The antioxidant activity of YHC-T-2414 was assessed by determining serum MDA levels, which are indicative of the antioxidant defense status. MDA is a final product of lipid peroxidation and is widely recognized as a representative marker of oxidative stress (42). Serum MDA levels were significantly reduced in all YHC-T-2414-treated groups compared with the normal control group, with reductions comparable to those observed in the creatine-treated positive control group (Figure 7A). In addition, to evaluate the anti-inflammatory effects of YHC-T-2414, serum levels of the inflammatory cytokines TNF-α and IL-6 were measured. Inflammation has been reported to contribute to fatigue through multiple mechanisms, including oxidative stress, impaired energy metabolism, and reduced muscle function (43, 44). Both serum TNF-α and IL-6 levels showed a decreasing trend following YHC-T-2414 administration, and a significant reduction compared with the control group was observed in the 25 mg/kg YHC-T-2414-treated group (Figures 7B,C). In summary, these findings suggest that YHC-T-2414 may exert antioxidant and anti-inflammatory effects, as evidenced by the reduced serum levels of MDA, TNF-α, and IL-6. These biological activities may contribute, at least in part, to its potential anti-fatigue effects.

Recent studies have reported that various natural products exhibit anti-fatigue effects through diverse biological mechanisms. Astaxanthin has been shown to alleviate chronic exercise-induced fatigue by reducing oxidative stress and improving mitochondrial function (45). Likewise, *Cordyceps* has been reported to improve exercise endurance and fatigue-related biomarkers through modulation of the NRF2/KEAP1/heme oxygenase-1 (HO-1) antioxidant signaling pathway (46). Similarly, *Schisandra chinensis* extract exerts anti-fatigue effects by regulating energy metabolism through the AMPK/PGC-1α pathway and antioxidant defense through the NRF2/HO-1 pathway (7). In addition, Spirulina-derived peptides have been reported to enhance exercise performance and improve serum fatigue-related biomarkers by strengthening antioxidant defense mechanisms (47). Consistent with these previous findings, YHC-T-2414 improved physical endurance while attenuating oxidative stress, inflammation, and fatigue-related biomarkers. Notably, the present study further suggests that these beneficial effects may be associated with coordinated regulation of the AMPK-NRF2-KEAP1 and AMPK-SIRT1-PGC-1α signaling pathways, thereby providing mechanistic evidence for the anti-fatigue activity of the whole *H. pluvialis* extract, rather than that of a purified single component.

The present study provides evidence that YHC-T-2414 may exert anti-fatigue effects in both *in vitro* and *in vivo* models. In H_2_O_2_-treated C2C12 myoblasts, YHC-T-2414 mitigated cell viability, reduced LDH and CK activities, and showed antioxidant activity, as evidenced by increased DPPH and ABTS radical scavenging activities and reduced intracellular ROS levels. In the weight-loaded forced swimming mouse model, YHC-T-2414 prolonged exhaustion swimming time and improved fatigue-related biochemical markers, including lactate, CK, LDH, MDA, TNF-α, and IL-6. These beneficial effects may be associated with modulation of the AMPK–NRF2–KEAP1 signaling pathway involved in antioxidant defense and the AMPK–SIRT1–PGC-1α signaling pathway associated with mitochondrial biogenesis. Although these molecular mechanisms were demonstrated only in C2C12 myoblasts, the *in vitro* findings may provide mechanistic support for the anti-fatigue effects observed *in vivo*. Therefore, YHC-T-2414 may have potential as a natural functional ingredient for alleviating exercise-induced fatigue. However, further studies are needed to confirm whether these signaling pathways are similarly regulated in skeletal muscle following YHC-T-2414 administration.

## Data Availability

The original contributions presented in this study are included in this article/supplementary material, further inquiries can be directed to the corresponding author.
